# Exploring the Antibiotic Production Potential of Heterotrophic Bacterial Communities Isolated from the Marine Sponges *Crateromorpha meyeri*, *Pseudaxinella reticulata*, *Farrea similaris*, and *Caulophacus arcticus* through Synergistic Metabolomic and Genomic Analyses

**DOI:** 10.3390/md20070463

**Published:** 2022-07-20

**Authors:** Sanaullah Tareen, Peter J. Schupp, Naveed Iqbal, Joachim Wink

**Affiliations:** 1 Microbial Strain Collection (MISG), Helmholtz Centre for Infection Research (HZI), 38124 Braunschweig, Germany; sanaullah.tareen@helmholtz-hzi.de; 2 Department of Microbiology, Balochistan University of Information Technology Engineering and Management Sciences, Quetta 87300, Pakistan; 3 Institute for Chemistry and Biology of the Marine Environment, Oldenburg University, 26129 Oldenburg, Germany; peter.schupp@uni-oldenburg.de; 4 Department of Biotechnology, Balochistan University of Information Technology Engineering and Management Sciences, Quetta 87300, Pakistan; naveed.iqbal@buitms.edu.pk

**Keywords:** marine sponge, antimicrobials, fatty acids, phylogenetic analyses, *Qipengyuania*

## Abstract

The discovery of novel secondary metabolites is actively being pursued in new ecosystems. Sponge-associated bacteria have been in the limelight in recent years on account of their ability to produce bioactive compounds. In this study, heterotrophic bacteria associated with four sponge species were isolated, taxonomically identified, and subjected to screening for the production of bioactive entities against a panel of nine microorganisms, including Gram-positive and negative bacteria, as well as yeast and fungi. Of the 105 isolated strains, 66% were represented by *Proteobacteria*, 16% by *Bacteriodetes*, 7% by *Actinobacteria*, and 11% by *Firmicutes*. Bioactivity screening revealed that 40% of the total isolated strains showed antimicrobial activity against one or more of the target microorganisms tested. Further, active extracts from selective species were narrowed down by bioassay-guided fractionation and subsequently identified by HR-ESI-MS analyses to locate the active peaks. Presumably responsible compounds for the observed bioactivities were identified as pentadecenoic acid, oleic acid, and palmitoleic acid. One isolate, *Qipengyuania pacifica* NZ-96^T^, based on 16S rRNA novelty, was subjected to comparative metabolic reconstruction analysis with its closest phylogenetic neighbors, revealing 79 unique functional roles in the novel isolate. In addition, genome mining of *Qipengyuania pacifica* NZ-96^T^ revealed three biosynthetic gene clusters responsible for the biosynthesis of terpene, beta lactone, lasso peptide, and hserlactone secondary metabolites. Our results demonstrate the ability to target the sponge microbiome as a potential source of novel microbial life with biotechnological potential.

## 1. Introduction

The marine environment, one of the most untapped biological reservoirs, carries tremendous importance in terms of its rich biological and chemical diversity [1]. Marine sponges (phylum *Porifera*) are filter-feeding sedentary organisms that harbor diverse communities of microorganisms, representing 50–60% of the total sponge biomass and densities of 10^8^ to 10^10^ bacteria per gram of sponge wet weight [2,3]. Marine sponges have a global distribution and are crucial members of marine communities. Comparatively, they are more diverse and numerous than other marine organisms, and even the sponge species diversity outpaces the combined species diversity of other marine organisms [4]. Sponges have been in the limelight for researchers for quite some time due to the production of biologically active secondary metabolites. These compounds are critical in host defense against predators, thus playing a major role in sponge survival in the marine environment [5].

The biological activity of sponge-derived natural products is astonishing and includes anti-tumor, anti-cancer, anti-malarial, anti-inflammatory, cytotoxic, photoprotective, antibiotic, and antifouling properties [6]. So far, clinically relevant bioactive products from sponge microbial symbionts have been tracked down from geographically different regions, such as the South China Sea, Mediterranean Sea, Indonesia, Great Barrier Reef of Australia, Papua New Guinea, and the Indo-Pacific region. An ample number of bioactive compounds have been isolated from marine sponge symbionts since the discovery of spongothymidine and spongouridine from the marine sponge *Cryptotethya crypta* in the early 1950s [7]. Recently, a surge has been seen in the discovery of new bioactive compounds from the sponge, with about 300 novel compounds isolated only from phylum *Porifera*, and these have been subjected to clinical and pre-clinical trials [8]. The dominant phyla as producers of novel bioactive compounds are *Actinobacteria, Proteobacteria, Cyanobacteria, Bacteroides*, and *Firmicutes* [9]. Additionally, numerous other bacterial phyla are also present in the sponge microbial community, including *Chloroflexi*, *Acidobacteria*, *Poribacteria*, *Deinococcus-Thermus*, *Gemmatimonadetes*, *Nitrospira*, *Planctomycetes*, and *Spirochaetes* as revealed mainly by culture-independent phylogenetic analysis [10].

The present work was carried out to investigate the culturable bacterial diversity associated with four marine sponges, namely, *Crateromorpha meyeri*, *Pseudaxinella reticulata*, *Farrea similaris*, and *Caulophacus arcticus*, and to demonstrate their ability to produce antimicrobial compounds. *Pseudaxinella reticulata* belong to the class *Demospongiae*, while the other three sponges belong to the class *Hexactinellida*. In 1872, J. E. Gray first discovered *Crateromorpha meyeri*, while *Pseudaxinella reticulata* was discovered by Ridley and Dendy in 1886 [11,12]. The genus *Farrea* was first described by Bowerbank in 1862, and the species *Farrea similaris* was described in 2011 by Reiswig and Kelly [13]. *Caulophacus* was first described by Schulze in 1886 during a voyage of the H.M.S. Challenger during the years 1873–1876, with the species *Caulophacus arcticus* being described in 1885 by Hansen [14]. The marine sponge *Pseudaxinella reticulata* is already known for its broad-spectrum antifungal activity exhibited by its guanidine-containing natural products [15]. Bacteria associated with these four sponge species have not been investigated. Herein, we report their culturable bacterial diversity and its antimicrobial potential against a panel of pathogenic and environmental microorganisms for the first time.

## 2. Results and Discussion

### 2.1. Isolation and Phylogenetic Analysis of Sponge-Associated Bacteria

**Figure 1:**
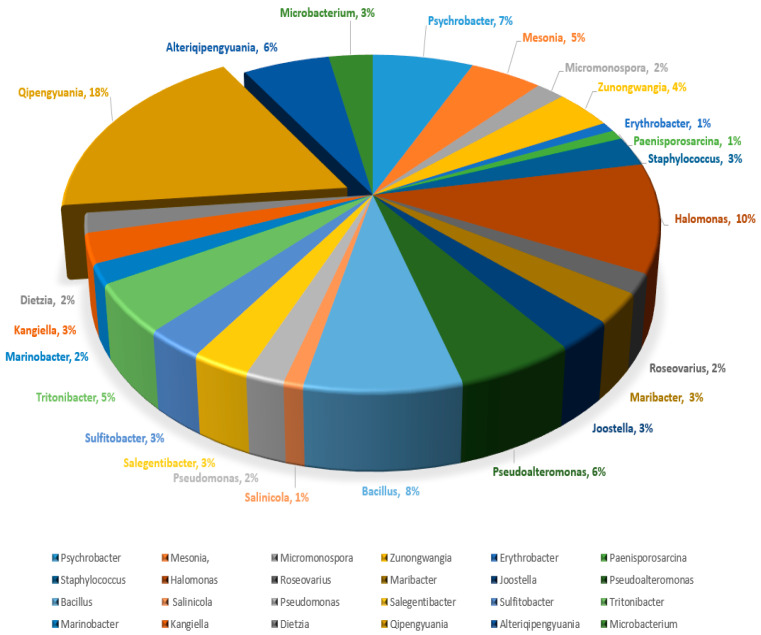
Percentage composition of isolated sponge-associated bacterial genera.

A total of 105 bacterial strains were isolated from four marine sponges (*Crateromorpha meyeri*, *Pseudaxinella reticulata*, *Farrea similaris*, and *Caulophacus arcticus*). All assayed media allowed the isolation of bacterial strains; however, marine agar medium provided the greatest number of isolates and broadest diversity from the four sponge samples. The isolates were distributed over 24 different genera and included *Psychrobacter*, *Mesonia*, *Micromonospora*, *Zunongwangia*, *Erythrobacter*, *Paenisporosarcina*, *Staphylococcus*, *Halomonas*, *Roseovarius*, *Maribacter*, *Joostella*, *Pseudoalteromonas*, *Bacillus*, *Salinicola*, *Pseudomonas*, *Alteriqipengyuania*, *Salegentibacter*, *Microbacterium*, *Sulfitobacter*, *Tritonibacter*, *Marinobacter*, *Qipengyuania*, *Kangiella*, and *Dietzia*. The dominant genus was *Qipengyuania* with 18% of the total sponge isolates comprising its members, followed by *Halomonas* with 10% of the total isolates (Figure 1). The isolated strains shared a similarity of 98% to 100% with their closest phylogenetic neighbors over a region of more than 1300 bp and belonged to the *Bacteroidetes*, *Actinobacteria*, *Firmicutes*, and *Proteobacteria* (*Alphaproteobacteria* and *Gammaproteobacteria*) phyla. Within *Proteobacteria*, the most abundant class was *Alphaproteobacteria* with 37 isolates, and *Gammaproteobacteria* was represented by 32 isolates. *Bacteroidetes*, *Actinobacteria*, and *Firmicutes* were represented by 17, 7, and 12 isolates, respectively (Table 1).

### 2.2. Antimicrobial Activity Assessment Based on a Minimum Inhibitory Concentration Assay

**Figure 2:**
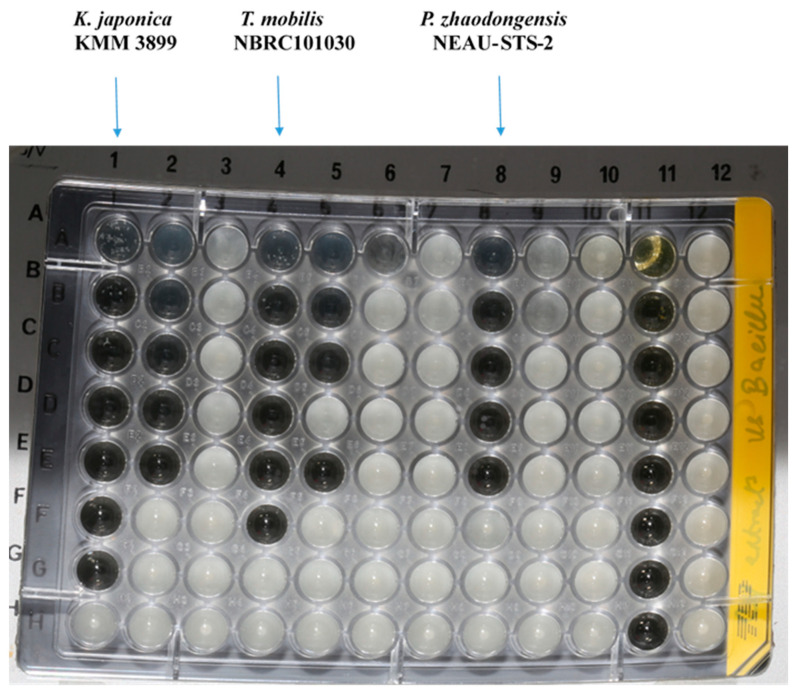
Minimum inhibitory concentration assay of *Kangiella*, *Tritonibacter*, and *Pseudomonas* against *Bacillus subtilis* DSM 10, displaying potent activity.

The sponge isolates capability of producing antimicrobial entities against nine indicator microorganisms, including Gram-negative (*Pseudomonas aeruginosa* PA14, *Escherichia coli* acrB JW25113, and *Citrobacter freundii* DSM 30039^T^) and Gram-positive bacteria (*Bacillus subtilis* DSM 10^T^, *Staphylococcus aureus* (Newman), and *Mycobacterium smegmatis* ATCC 700084), as well as yeast and fungi (*Candida albicans* DSM 1665, *Mucor hiemalis* DSM 2656^T^, and *Pichia anomala* DSM 6766), was tested by using the minimum inhibitory concentration (MIC) assay. Crude extracts of all isolated species were obtained by cultivating them in a marine broth (MB) medium. Of the total bacteria (105) screened for antimicrobial activity, 40% (42 isolates) of the total isolated bacteria showed antimicrobial activity against one or more of the target microorganisms tested. The highest number of active hits was found in *Gammaproteobacteria* (54.7%), followed by *Bacteriodetes* (23.8%) and *Alphaproteobacteria* (21.4%). Of the eleven genera showing antimicrobial activity, *Halomonas*, *Pseudoalteromonas*, *Tritonibacter*, and *Kangiella* presented the highest positive hits. The bioactive strains displayed weak to moderate activity against *Bacillus subtilis* DSM 10, *Staphylococcus aureus* (Newman), *Candida albicans* DSM 1665, and *Mucor hiemalis* DSM 2656. None of the isolates produced effective antimicrobial compounds against *Pseudomonas aeruginosa* PA14, *Escherichia coli* DSM 1116, *Citrobacter freundii* DSM 30039^T^, and *Acinetobacter baumannii* DSM 30008. *Maribacter* and *Zunongwangia* were the only genera effective against *Mycobacterium smegmatis* ATCC 700084, whereas *Halomonas* and *Zunongwangia* showed weak activity against *Mucor hiemalis* DSM 2656. *Kangiella*, *Tritonibacter*, and *Pseudomonas* displayed potent activity against *Bacillus subtilis* DSM 10 (Figure 2) and *S. aureus* Newman; thus, they were selected for bioassay-guided fractionation to locate the peaks related to the activity. The highest activity was observed for the crude extract of *Kangiella japonica* KMM 3899, with MIC values of 1.04 and 8.3 µg/mL against *B. subtilis* DSM 10 and *S. aureus* Newman, respectively, followed by *Tritonibacter mobilis* NBRC101030 with MIC values of 2.08 and 33.3 µg/mL against *B. subtilis* and *S. aureus*, respectively (Table 2). Numerous bioactive genera isolated in this study were found to possess antimicrobial activity. *Pseudoalteromonas* is a good example of a well-known bioactive compound producer [16,17], as is *Pseudomonas* [17,18]. Bioactivity has also been displayed in the *Marinobacter* [19] and *Halomonas* genera [20,21].

### 2.3. Bioassay-Guided Fractionation and Mass Spectrometric Analysis

**Figure 3:**
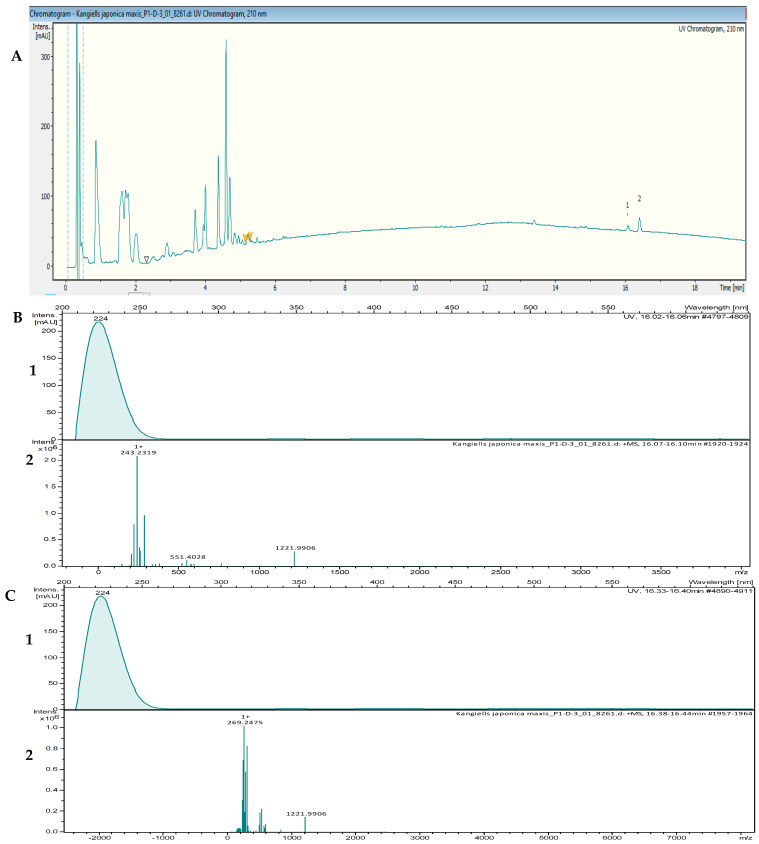
(**A**) UV chromatogram from a mass spectrometry run of a crude extract of *Kangiella japonica* KMM 3899. (**B**) HR-ESI-MS data of the targeted peak: (1) UV absorption band at 224 nm; (2) mass spectrum at the retention time of *t*_R_ = 16.08 min, *m*/*z* 243.2319 [M + H]^+^. (**C**) (1) UV absorption band at 224 nm; (2) mass spectrum at the retention time of *t*_R_ = 16.40 min, *m*/*z* 269.2475 [M + H]^+^.

The crude extracts with the highest activity were fractionated by using reversed-phase high-performance liquid chromatography (RP-HPLC) and were tested in bioassays against *B. subtilis* DSM 10 and *S. aureus* Newman to determine the peak-related activities. The micro-fractionation was performed in 96-well plates to target the active compounds of the crude extract. The HPLC fractionation experiment from *Kangiella japonica* KMM 3899 resulted in two fractions that inhibited the growth of *B. subtilis* DSM 10. To identify the associated target masses of the detected active fractions, the crude extract was analyzed by using high-resolution electrospray ionization–mass spectrometry (HR-ESI-MS). The two peaks, which were correlated with activity in the MS chromatogram (Figure 3A), were detected in the HR-ESI-MS total ion current (TIC) and were named compounds **1** and **2**. The mass spectra of compound **1**, with the retention time (*t*_R_) = 16.08 min, showed ions at mass-to-charge ratios (*m*/*z*) of 225.2211 *m*/*z*, 243.2319 *m*/*z*, and 265.2139 *m*/*z*, corresponding to [M − H_2_O + H]^+^, [M + H]^+^, and [M + Na]^+^, respectively. The UV absorption bands were located at 224 nm (Figure 3B). The second compound, with a chromatographic retention time of 16.43 min, presented mass spectra with ions with 251.2369 *m*/*z*, 269.2475 *m*/*z*, 291.2295 *m*/*z*, and 537.4882 *m*/*z*, corresponding to [M − H_2_O + H]^+^, [M + H]^+^ [M + Na]^+^, and [2M + H]^+^, respectively. The UV absorption bands were located at 224 nm (Figure 3C). The suggested formulas of C_15_H_30_O_2_ and C_17_H_32_O_2_ were deduced for compounds **1** and **2** by using the SmartFormula™ algorithm in ESI Compass Data Analysis from Bruker version 5.3. A search in the Dictionary of Natural Products (DNP) database revealed that the protonated ions of 243.2319 *m*/*z* ([M + H]^+^) and 269.2475 *m*/*z* ([M + H]^+^) were tentatively identified as pentadecanoic acid ([M + H]^+^) 243.22458 *m*/*z* and a molecular formula of C_15_H_30_O_2_) (Figure S1) and 9-hexadecenoic acid methyl ester ([M + H]^+^ 269.24023 *m*/*z* and a molecular formula of C_17_H_32_O_2_ (Figure S2), respectively. The latter compound was also called methyl palmitelaidate.

**Figure 4:**
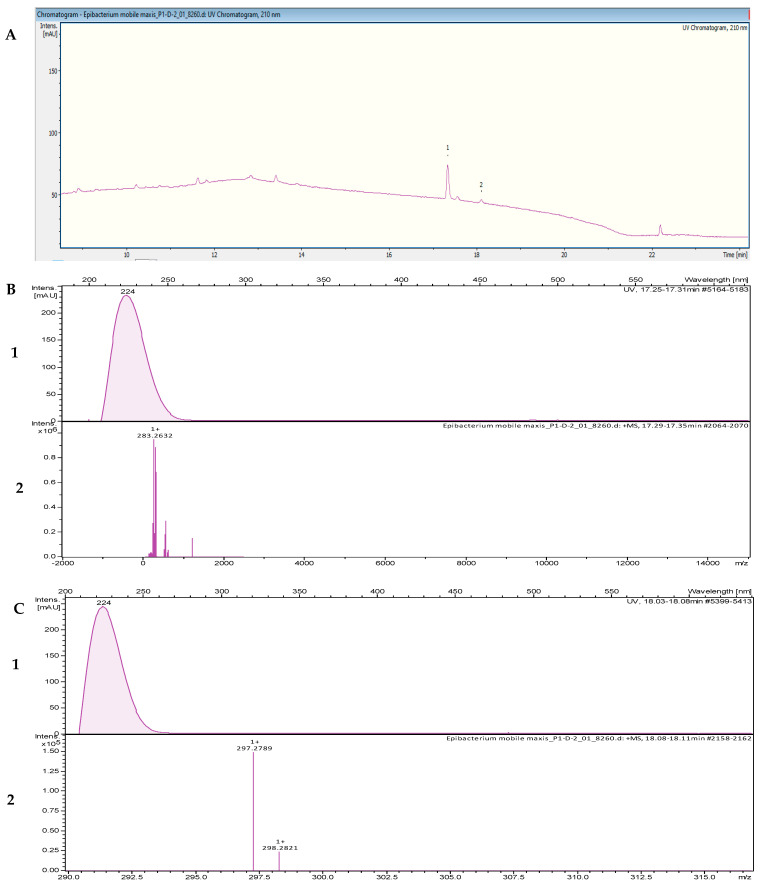
(**A**) UV chromatogram from a mass spectrometry run of a crude extract of *Tritonibacter mobilis* NBRC101030. (**B**) HR-ESI-MS data of the targeted peak: (1) UV absorption band at 224 nm; (2) mass spectrum at the retention time of *t*_R_ = 17.31 min, *m*/*z* 283.2632 [M + H]^+^. (**C**) (1) UV absorption band at 224 nm; (2) mass spectrum at the retention time of *t*_R_ = 18.09 min, *m*/*z* 297.2789 [M + H]^+^.

The fractionation of the crude extract of *Tritonibacter mobilis* NBRC101030 (also called *Epibacterium mobile*) yielded two fractions that inhibited the growth of *B. subtilis* DSM 10. High-resolution electrospray ionization–mass spectrometry (HR-ESI-MS) analyses of a bioactive methanolic extract were carried out to identify the active fractions. The two peaks, which were correlated with activity in the MS chromatogram (Figure 4A), were detected in the HR-ESI-MS total ion current (TIC) and named compounds **1** and **2**. The peak of compound **1** in the base peak chromatogram with the retention time (*t*_R_) = 17.35 min showed ions at a mass-to-charge ratio (*m*/*z*) of 265.2526 *m*/*z*, 283.2632 *m*/*z*, 305.2451 *m*/*z*, and 565.5194 *m*/*z*, corresponding to [M − H_2_O + H]^+^, [M + H]^+^, [M + Na]^+^, and [2M + H]^+^, respectively. The UV absorption bands were located at 200 and 224 nm (Figure 4B). The second compound, with a chromatographic retention time of 18.09 min, displayed mass spectra with ions with *m*/*z* 279.2682, *m*/*z* 297.2789, *m*/*z* 319.2608, and *m*/*z* 593.5500 corresponding to [M − H_2_O + H]^+^, [M + H]^+^, [M + Na]^+^, and [2M + H]^+^, respectively. The UV absorption bands were located at 224 nm (Figure 4C). The suggested formulas of C_18_H_34_O_2_ and C_19_H_36_O_2_ were obtained for compounds **1** and **2** with the SmartFormula™ algorithm in ESI Compass Data Analysis from Bruker. A search in the Dictionary of Natural Products (DNP) database revealed that the protonated ions *m*/*z* 283.2632 [M + H]^+^ and *m*/*z* 297.2789 [M + H]^+^ were tentatively identified as 9-octadecenoic acid ([M + H]^+^283.25588 *m*/*z* and a molecular formula of C_18_H_34_O_2_) (Figure S3) and methyl oleate, also called oleic acid methyl ester ([M + H]^+^297.27153 *m*/*z* and a molecular formula of C_19_H_36_O_2_; Figure S4), respectively.

**Figure 5:**
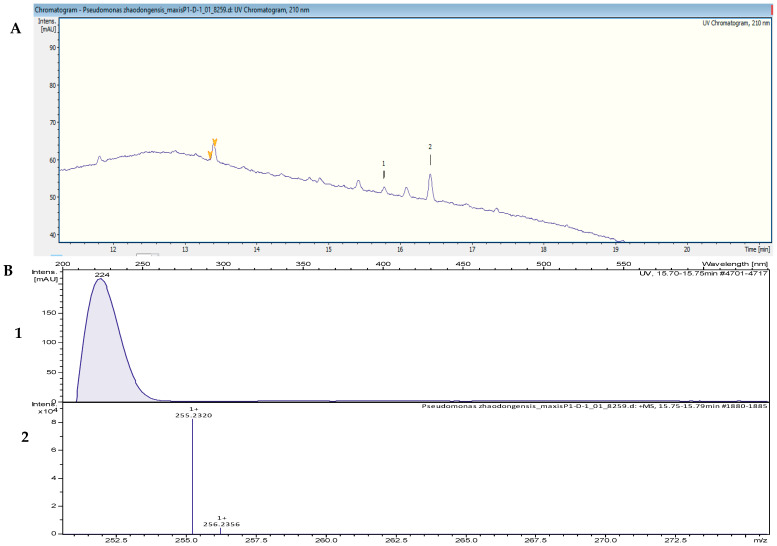
(**A**) UV chromatogram from a mass spectrometry run of a crude extract of *Pseudomonas zhaodongensis* NEAU-STS-21. (**B**) HR-ESI-MS data of the targeted peak: (1) UV absorption band at 224 nm; (2) mass spectrum at the retention time of *t*_R_ = 15.78 min, *m*/*z* 255.2320 [M + H]^+^.

**Figure 6:**
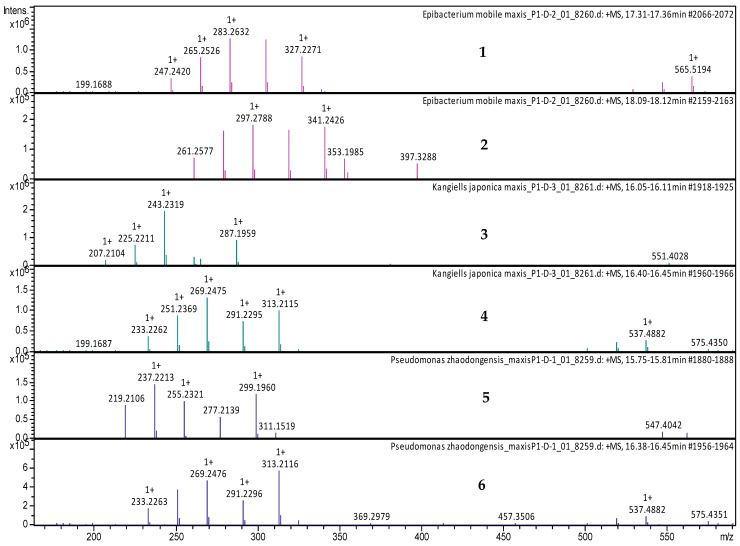
LC-MS data of the selected active extracts at 200–640 nm. (**1**) MS spectrum of *Tritonibacter mobilis* (also known as *Epibacterium mobile*) extract at *t*_R_ = 17.35 min, with mass-to-charge ratios (*m*/*z*) of 265.2526 *m*/*z*, 283.2632 *m*/*z*, 305.2451 *m*/*z*, and 565.5194 *m*/*z*, corresponding to [M − H_2_O + H]^+^, [M + H]^+^, [M + Na]^+^, and [2M + H]^+^, respectively. (**2**) MS spectrum of *Tritonibacter mobilis* (also known as *Epibacterium mobile*) at *t*_R_ = 18.09 min, with *m*/*z* 279.2682, *m*/*z* 297.2789, *m*/*z* 319.2608, and *m*/*z* 593.5500, corresponding to [M − H_2_O + H]^+^, [M + H]^+^, [M + Na]^+^, and [2M + H]^+^, respectively. (**3**) MS spectrum of *Kangiella japonica* at *t*_R_ = 16.10 min, with mass-to-charge ratios (*m*/*z*) of 225.2211 *m*/*z*, 243.2319 *m*/*z*, and 265.2139 *m*/*z*, corresponding to [M − H_2_O + H]^+^, [M + H]^+^, and [M + Na]^+^, respectively. (**4**) MS spectrum of *Kangiella japonica* at *t*_R_ = 16.40 min, with *m*/*z* 251.2369, *m*/*z* 269.2475, *m*/*z* 291.2295, and *m*/*z* 537.4882, corresponding to [M − H_2_O + H]^+^, [M + H]^+^, [M + Na]^+^, and [2M + H]^+^, respectively. (**5**) MS spectrum of *Pseudomonas zhaodongensis* at *t*_R_ = 15.75 min, with mass-to-charge ratios (*m*/*z*) of 237.2213 *m*/*z*, 255.2321 *m*/*z*, and 277.2149 *m*/*z*, corresponding to [M − H_2_O + H]^+^, [M + H]^+^, and [M + Na]^+^, respectively. (**6**) MS spectrum of *Pseudomonas zhaodongensis* at *t*_R_ = 16.40 min, with *m*/*z* 251.2369, *m*/*z* 269.2475, *m*/*z* 291.2295, and *m*/*z* 537.4882, corresponding to [M − H_2_O + H]^+^, [M + H]^+^, [M + Na]^+^, and [2M + H]^+^, respectively.

**Figure 7:**
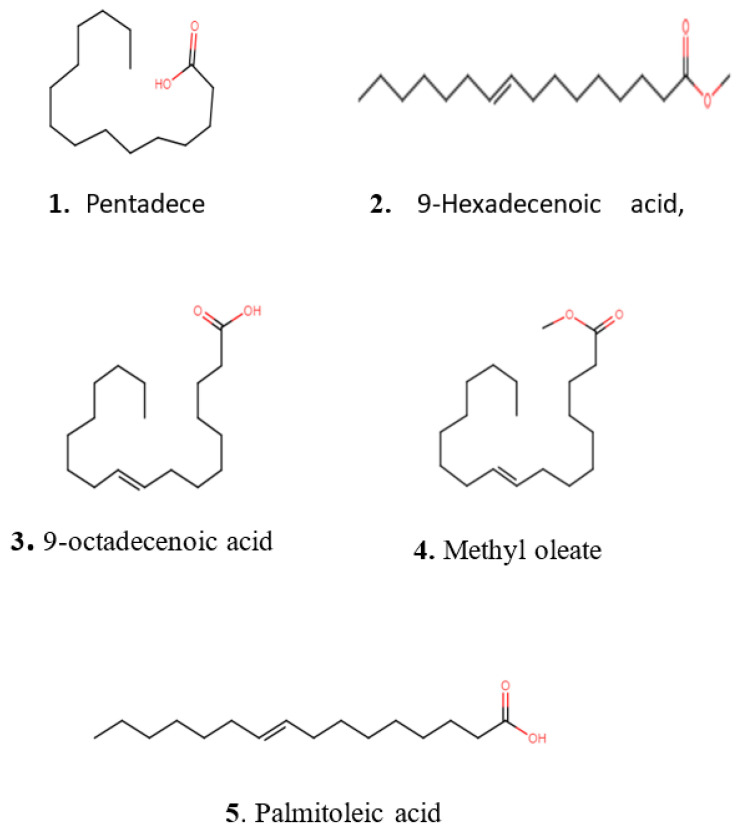
Chemical structures of representative secondary metabolites putatively identified with HR-ESI-MS in crude extracts of *Kangiella*, *Tritonibacter*, and *Pseudomonas* using the Dictionary of Natural Products database.

The HPLC fractionation experiment from *Pseudomonas zhaodongensis* NEAU-STS-21 resulted in three fractions that inhibited the growth of *S. aureus* Newman. Identification of the corresponding target masses of the active fractions obtained was carried out by using high-resolution electrospray ionization–mass spectrometry (HR-ESI-MS) analyses of the crude extract. Two of the active fractions corresponded to a single peak. The two peaks, which were correlated with activity in the UV chromatogram of the mass spectrometry run (Figure 5A), were detected in the HR-ESI-MS total ion current (TIC) and named compounds **1** and **2**. The mass spectra of compound **1**, with the retention time (*t*_R_) = 15.78 min, showed ions at a mass-to-charge ratio (*m*/*z*) of 237.2213 *m*/*z*, 255.2321 *m*/*z*, and 277.2149 *m*/*z* corresponding to [M − H_2_O + H]^+^, [M + H]^+^, and [M + Na]^+^, respectively. The UV absorption bands were located at 224 nm (Figure 5B). The second compound, with a chromatographic retention time of 16.43 min, presented mass spectra with ions with *m*/*z* 251.2369, *m*/*z* 269.2475, *m*/*z* 291.2295, and *m*/*z* 537.4882, corresponding to [M − H_2_O + H]^+^, [M + H]^+^, [M + Na]^+^, and [2M + H]^+^, respectively. The UV absorption bands were located at 224 nm. Using the SmartFormula™ algorithm in ESI Compass Data Analysis from Bruker, the suggested formulas of C_16_H_30_O_2_ and C_17_H_32_O_2_ were assigned to compounds **1** and **2**, respectively. The protonated ions with *m*/*z* 255.2321 [M + H]^+^ and *m*/*z* 269.2475 [M + H]^+^ were tentatively identified as palmitoleic acid ([M + H]^+^ 255.22458 *m*/*z* and molecular formula of C_16_H_30_O_2_) (Figure S5) and 9-hexadecenoic acid methyl ester ([M + H]^+^ 269.24023 *m*/*z* and a molecular formula of C_17_H_32_O_2_), respectively, as revealed by the Dictionary of Natural Products (DNP) database. The MS spectrum data of all of the selected active peaks in the range of 200–640 nm depicting [M − H_2_O + H]^+^, [M + H]^+^, [M + Na]^+^, and [2M + H]^+^ are shown in Figure 6. The chemical structures of the representative secondary metabolites in the crude extracts of *Kangiella*, *Tritonibacter*, and *Pseudomonas* that were putatively identified by HR-ESI-MS by using the Dictionary of Natural Products database are shown in Figure 7.

### 2.4. Thorough Functional Genome Annotation of a Novel Isolate of Qipengyuania pacifica NZ-96^T^

Of the total isolates, the species *Qipengyuania pacifica* NZ-96^T^ was found to be novel, exhibiting 98.3–98.8% 16S rRNA sequence homology with the closest phylogenetic members of the Gram-negative *Qipengyuania* genus [22]; thus, it was selected for whole-genome sequencing and comparative biosynthetic genomic architecture analysis with the closest neighbor species. Whole-genome sequencing is a key approach to revealing the microbial bioactive potential for encoding new NPs [23,24]. The contiguous draft genome of the *Q. pacifica* NZ-96^T^ consists of a single circular chromosome of 3,497,702 bp with a G+C content of 60.8%. The high-throughput genome annotation of *Q. pacifica* NZ-96^T^ using RAST revealed 54 contigs harboring 3494 coding DNA sequences (CDS) and 55 RNA-encoding genes (49 tRNA + 6 rRNA). For the whole genome of *Q. pacifica* NZ-96^T^, the RAST-based in-depth curation revealed significant enrichment of 877 functional roles using 973/3494 coding DNA sequences. All 877 functional roles identified were grouped into 282 functional sets (called a subsystem) [25] that were distributed among 27 core categories, where all functional roles belonging to a single subsystem implemented a specific biological process or structural complex. The genome mining of *Q. pacifica* NZ-96^T^ revealed 346 (*Q. pelagi*: 340; *Q. citrea*: 336) protein-encoding genes that enriched ten distinct metabolic functional categories of the subsystem. The enriched metabolism-associated subsystem categories were for protein, DNA, RNA, phosphorus, sulfur, nitrogen, potassium, aromatic compounds, secondary metabolites, and iron metabolisms. Among all of these, the protein metabolism was found to be the most enriched subsystem category in all three closely related bacterial species (gene count: NZ-96^T^: 190, *Q. pelagi*: 177; *Q. citrea*: 180; Table S1). Upon comparing with the closest 16S rRNA sequence-based phylogenetic strains, NZ-96^T^, *Q. pelagi* (JCM 17468), and *Q. citrea* (CGMCC 1.8703) harbored comparable counts of 44, 43, and 52 protein-encoding genes in a subsystem category associated with ‘virulence, disease, and defenses’ (Table S2). For *Q. pacifica* NZ-96^T^, the ‘resistance to antibiotics and toxic compounds’ category possessed eight subsystems. Of all eight, the subsystems conferring resistance to fluoroquinolones and beta-lactamase were found to be equally enriched by two (*gyrA*: DNA gyrase subunit A; *gyrB*: DNA gyrase subunit B) and one (*BLc*: Beta-lactamase class C) genes, respectively, in all three closely related *Qipengyuania* species (NZ-96^T^, *Q. pelagi*, and *Q. citrea*) [26,27].

### 2.5. Subsystem and Functional-Role-Based Comparative Genomics of Qipengyuania pacifica NZ-96^T^

High-throughput deep genome annotations using RAST were also performed for the two closely related *Qipengyuania* species to acquire subsystem enrichment data for a comparative metabolic reconstruction analysis among *Q. pacifica* NZ-96^T^, *Q. pelagi* UST081027-248^T^, and *Q. citreus* RE35F/1^T^.

**Figure 8:**
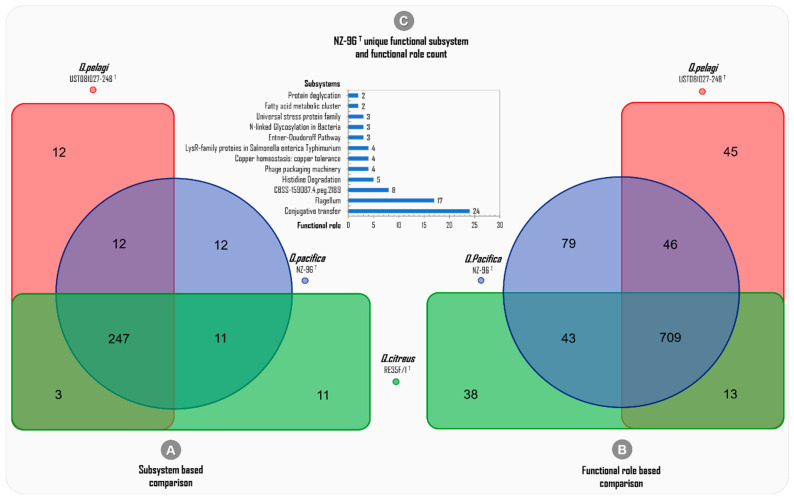
Deep-annotation-data-based genome-wide comparison among the subsystems and functional roles of the three phylogenetically related *Qipengyuania* species of *Qipengyuania pacifica* NZ-96^T^, *Qipengyuania pelagi* UST081027-248^T^, and *Qipengyuania citreus* RE35F/1^T^.

The subsystem-based genome-wide comparison among the three *Qipengyuania* species included revealed 247 commonly shared subsystems, whereas only 12, 11, and 12 subsystems were uniquely attributed to *Q. pacifica* NZ-96^T^, *Q. citreus* RE35F/1^T^, and *Q. pelagi* UST081027-248^T^, respectively (Figure 8A).

Since a subsystem is a set of functional roles that execute a specific biological process [25], to reveal the unique features of *Q. pacifica* NZ-96^T^, the genome-wide comparison among the closely related *Qipengyuania* species was further curated to the ‘functional roles’ level. 

The deep annotation predicted a total of 2493 (973 distinctive) ‘functional roles’ across the three whole genomes of *Q. pacifica* NZ-96^T^ (877), *Q. citreus* (803), and *Q. pelagi* (877) (Table 3). The functional-role-based comparison showed 709 ‘common functional roles’ being mutually harbored among the three genomes included, and the highest number of ‘unique functional roles’ was predicted for *Q. pacifica* NZ-96^T^ (79) compared to *Q. pelagi* UST081027-248^T^ (45) and *Q. citreus* RE35F/1^T^(38), as shown in Figure 8B. This higher level of diversity of functional roles of *Q. pacifica* NZ-96^T^ can be attributed to the differences observed at the 16S rRNA, dDDH, and ANI levels. In this study, for *Q. pacifica* NZ-96^T^, the numbers of unique functional roles discovered, which were found to be absent in the genomes of *Q. pelagi* and *Q. citreus*—are 24 in conjugative transfer, 17 in the flagellum, 8 in CBSS-159087.4.peg.2189, 5 in histidine degradation, 4 in phage packaging machinery, 4 in copper homeostasis/copper tolerance, 4 in LysR-family proteins in *Salmonella enterica Typhimurium*, 3 in the Entner–Doudoroff pathway, 3 in N-linked glycosylation in bacteria, 3 in the universal stress protein family, 2 in the fatty acid metabolic cluster, and 2 in protein deglycation unique subsystems. The clustered bar in Figure 8C presents the total number of ‘unique functional roles’ and their respective subsystems discovered in *Q. pacifica* NZ-96^T^. 

### 2.6. Discovering the Genomic Potential of Qipengyuania pacifica NZ-96^T^ for Encoding Bioactive Secondary Metabolites

Microbial secondary metabolites serve as rich sources of bioactive compounds with potential applications as antibiotics or pharmaceutical drugs [28]. Studies have revealed that sets of two or more closely situated clusters of genes encode secondary metabolites. These clustered genes that together encode a secondary metabolite in a biosynthetic pathway are known as a biosynthetic gene cluster (BGC) [29]. To discover the secondary metabolome richness of NZ-96^T^, the whole genome sequence was scanned for the discovery of potential secondary metabolites encoding BGCs using anti-SMASH version 6.0 [30]. The sequence-similarity-based analysis found 80 different secondary-metabolite-associated genes clustered in three putative BGCs within the whole NZ-96^T^ genome, including betalactone, terpene, and hserlactone/lassopeptide. This finding suggests that *Qipengyuania pacifica* NZ-96^T^ has the genomic potential to encode at least three different bioactive secondary metabolites (Table 4). 

Among the three BGCs, cluster 1 (designated as NZ-96^T^ beta-lactone-like gene cluster) showed high gene-level sequence similarity with beta-lactone-dependent AMP synthase and ligase (pHMM detection rule used: beta lactone HMGL-like and AMP-binding); hence, it was proposed to be involved in beta lactone biosynthesis. For the beta-lactone-like gene cluster, a total of 31 genes were predicted, including two (i) core biosynthetic genes (Qpaβlact-G: *AMP-dependent synthetase* and Qpaβlact-N: *2-isopropylmalate synthase*), (ii) four additional biosynthetic genes (Qpaβlact-B: *AMP-dependent-ligases*, Qpaβlact-H: *haloalkane-dehalogenase*, Qpaβlact-K: *aldehyde-dehydrogenase*, and Qpaβlact-R: *pyridine-nucleotide-disulfide-oxidoreductase*), (iii) one regulatory gene (Qpaβlact-A: *TetR family transcriptional regulator*), (iv) one transport-related receptor gene (Qpaβlact-X: *TonB-dependent receptor*), and (v) the remaining twenty-three uncharacterized genes. 

**Figure 9:**
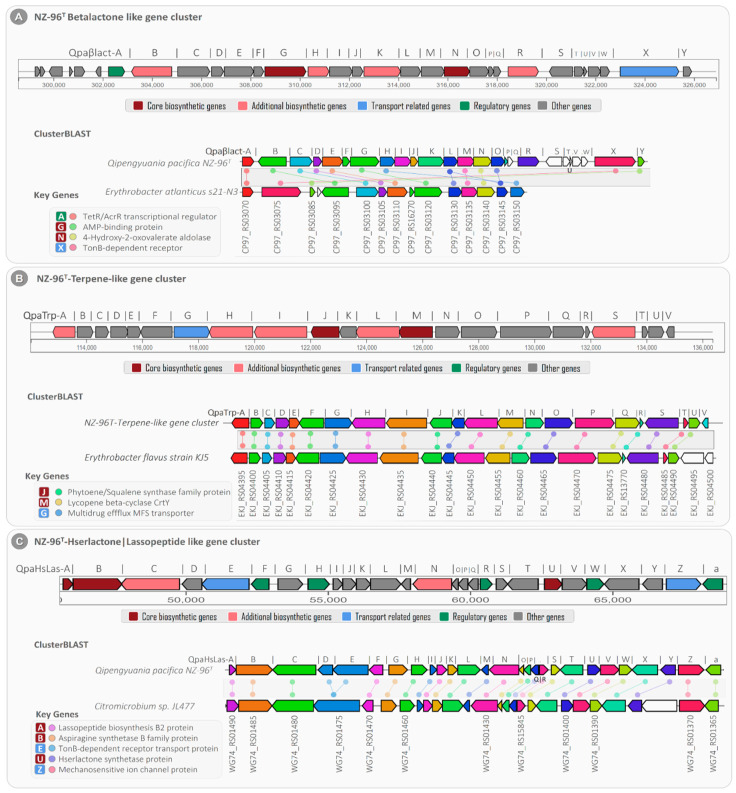
Clusters from the anti-SMASH database and other clusters of interest that are similar to the current region. Genes marked with the same color are interrelated. White genes have no relationship.

The detailed analysis revealed four of the twenty-three total uncharacterized genes with transposase activities, of which the Qpaβlact-b and Qpaβlact-d genes were found upstream and the Qpaβlact-S and Qpaβlact-V genes were located downstream from the core and additional biosynthetic clustered genes, thus suggesting their role in the horizontal gene transfer of the beta lactone BGC. The basic local alignment of the NZ-96^T^ beta-lactone-like gene cluster showed an average sequence similarity of ~67.6% with the fourteen clustered genes of *Erythrobacter atlanticus* s21-N3 (Figure 9A). 

The core biosynthetic genes Qpaβlact-G and Qpaβlact-N showed high sequence similarity with the *Erythrobacter atlanticus* s21-N3 AMP-binding protein (identity/coverage; 73%/97%) and 4-hydroxy-2-oxovalerate aldolase (similarity/coverage; 76%/99%), respectively (Table S3). Furthermore, the *TetR family transcriptional regulator* (Qpaβlact-A) and *TonB-dependent receptor* (Qpaβlact-X) genes were strongly correlated with TetR/AcrR family transcriptional regulator (similarity/coverage; 82.4%/98.6%) and TonB-dependent receptor (similarity/coverage; 80.2%/98.5%) encoded by *Erythrobacter atlanticus* s21-N3, respectively.

In addition to the NZ-96^T^ bet-lactone-like gene cluster, the NZ-96^T^ cluster-2 showed 66% sequence homology with the previously known terpene biosynthetic gene cluster (BGC0000656) encoded in the *Xanthobacter autotrophicus* Py2 genome [31]. Since cluster 2 consisted of twenty-one closely situated genes for the biosynthesis of zeaxanthin (terpene), it was called the NZ-96^T^ terpene-like gene cluster (Figure 9B). Of the total twenty-one genes, the NZ-96^T^ terpene-like gene cluster contained (i) two core biosynthetic genes (QpaTrp-J: *Phytoene synthase* and QpaTrp-M: *Lycopene cyclase*), (ii) five additional biosynthetic genes (QpaTrp-A: *SDR family oxidorductase*, QpaTrp-H: *methylmalonyl-CoA carboxyl transferase*, QpaTrp-I: *acetyl-CoA carboxylase*, QpaTrp-L: *phytoene desaturase*, and QpaTrp-S: *alanine symporter family protein*), (iii) one transporter gene (QapTrp-G: *multidrug efflux MFS transporter*), and fourteen additional genes. The basic local alignment search for the NZ-96^T^ terpene-like gene cluster displayed 91.2% sequence similarity with 21 genes of the terpene-encoding BGC (NZ_AP019389) of the *Erythrobacter flavus* strain KJ5 (Table S4). The core biosynthetic genes QpaTrp-J and QpaTrp-M exhibited 96% and 99% sequence identity with the *Phytoene synthase* (EKJ_RS04440) and *Lycopene cyclase* (EKJ_RS04455) genes of the *Erythrobacter flavus* strain KJ5, respectively. 

Cluster 3 from NZ-96^T^ consisted of a total of 27 clustered genes that showed high gene-level sequence similarity with the known hybrid Hserlactone|Lasoopeptide gene cluster. Based on this sequence similarity, the predicted cluster was designated as the NZ96^T^ Hserlactone|Lassopeptide- like gene cluster. This BGC contained (a) three core biosynthetic genes, (b) two additional biosynthetic genes, (c) two transport genes, (d) five regulatory genes, and (e) 15 other genes (Figure 9C). The core biosynthetic genes of this cluster were associated with lassopeptide biosynthesis B2 protein (QpaHsLas-A), asparagine synthetase B family protein (QpaHsLas-B), and Hse lactone synthetase protein (QpaHsLas-U). The additional biosynthetic genes displayed homologies with Atxe 2 family lassopeptide isopeptidase (QpaHsLas-C) and NADP-dependent oxidoreductases (QpaHsLas-N). In addition, based on the sequence similarity, the transport-related genes were related to TonB-dependent receptor transport protein (QpaHsLas-E) and mechanosensitive ion channel protein (QpaHsLas-Z) for extracellular transportation of Hserlactone and Lasoopeptide, respectively. The clusterBLAST analysis of the NZ96^T^ Hserlactone|Lassopeptide-like gene cluster exhibited ~92% gene-level sequence similarity with 26 genes of the Hserlactone|Lassopeptide gene cluster (NZ_CP011344) encoded by *Citromicrobium* sp. *JL477* (Figure 9C ClusterBLAST). The core biosynthetic genes Qpaβlact-A, Qpaβlact-B, and Qpaβlact-U showed sequence similarity with the *Citromicrobium* sp. *JL477* lassopeptide biosynthetase (identity/coverage; 92%/100%), asparagine synthetase (identity/coverage; 78%/99.8%), and Hserlactone synthetase (identity/coverage; 100%/100%), respectively (Table S5).

## 3. Materials and Methods

### 3.1. Sponge Sample Collection

The four marine sponges (*Crateromorpha meyeri*, *Pseudaxinella reticulata*, *Farrea similaris*, and *Caulophacus arcticus*) were collected in February 2017 during a sample collection expedition in a German research vessel Sonne Cruise SO254 (SONNE) using a remotely operated vehicle (ROV). Sampling was executed between 0.5 and 1.5 km below the ocean surface in muddy sediments with boulders and old chimneys in the Otago/Canterbury Slope (45°02′ N, 171°90′ W), Pacific Ocean, New Zealand. Samples were cryopreserved and transported to the microbial strain collection group in the Helmholtz Center for Infection Research, Braunschweig, Germany, where the sponge samples were kept at –80 °C until further processing. Isolated samples were identified by using morphological analysis of the shape, size, and skeletal arrangement according to the *Porifera* classification system [32].

### 3.2. Bacterial Culturing 

The sponge samples for bacterial isolation were rinsed with sterile artificial seawater (ASW) several times to remove the loosely bound bacterial cells, as well as debris. Approximately 1 cm^3^ of the sponge tissue samples were cut with a sterile scalpel, ground using a mortar and pestle, and immediately transferred to 9 mL of sterile artificial seawater. Several dilutions (10^−2^ to 10^−6^) were prepared, and 0.1 mL was spread onto several solid marine media, including a seawater glutamate (SWG) medium containing artificial seawater (3.9% (*w*/*v*) of sea salt from ATI Coral Ocean) and 0.1% sodium glutamate, solidified with 1.6% agar (Difco) [33], a marine agar (MA) 2216 medium (Difco Laboratories, NJ, BD, USA), and an ASW-CY medium (Casitone 3.00 g, CaCl_2_ × 2H_2_O 1.36 g, yeast extract 1.00 g, agar 16.00 g, distilled water 1000 mL, pH 7.2) supplemented with 39 g of artificial sea salts, as well as a VY/2 medium containing 39 g of artificial sea salts, 10 mL of baker’s yeast, 11.9 g of Hepes, 1 g of CaCl_2_ × 2H_2_O, 1 mL of vitamin B12 (0.5 mg/mL), 16.00 g of agar, and 1000 mL of distilled water, with a pH of 7.2 (the vitamin was added to the media after autoclaving). Filter-sterilized cycloheximide (50 mg/L) was added to the media after autoclaving to inhibit fungal growth. Agar plates were incubated at 30 °C and routinely checked for observable growth under a stereomicroscope for 2–4 weeks. The isolated strains were purified through repeated streaking on marine agar (MA, Difco). For further processing, purified strains were preserved in marine broth 2216 (MB, Difco Laboratories, BD, USA) supplemented with 50% (*v*/*v*) glycerol at −80 °C.

### 3.3. Phylogenetic Analysis of Isolated Strains

Molecular taxonomic characterization was carried out using 16S rRNA gene sequencing. Genomic DNA extraction from isolated strains was performed using the Nucleospin microbial DNA kit (Macherey-Nagel, Düren, Germany). A single colony was cultured in 10 mL of MB media and incubated on a rotary shaker (160 rpm) at 30 °C for 3 days. DNA extraction was performed according to the manufacturer’s instructions. Amplification of 16S rRNA gene fragments was carried out using the universal primers 27F (5-AGAGTTTGATCMTGGCTCAG-3) and 1492R (5-TACGGTTACCTTGTTACGACTT-3), yielding fragments of approximately 1450 bp in length [34]. PCR amplification was performed in 25 μL volumes that consisted of 12.5 μL of Jumpstart Taq ready mix (Sigma Aldrich, MD, USA), 1 μL each of forward and reverse primer (10 μM), 1 μL of template DNA, and 9.5 μL of PCR water. The JSRM was a combination of 99% pure deoxynucleotides, JumpStart Taq DNA polymerase, and buffers in an optimized reaction concentration. The PCR reaction was carried out in a Mastercycler Gradient (Eppendorf, Hamburg, Germany) with the following conditions: initial denaturation at 95 °C for 5 min, 34 cycles of denaturing at 94 °C for 0.5 min, annealing at 52 °C for 0.5 min, elongation at 72 °C for 2 min, and final elongation at 72 °C for 10 min. Following analysis through gel electrophoresis, the PCR products were purified using the Nucleospin gel and PCR clean-up kit (Macherey Nagel, Duren, Germany) as per the manufacturer’s instructions. DNA sequencing was performed by using a 96-capillary system from Applied Biosystems (ABI; 3730 xl DNA Analyzer). The F1100 (5-CAACGAGCGCAACCC-3), R1100 (5-GGGTTGCGCTCGTTG-3), and R518 (5-CGTATTACCGCGGCTGCTGG-3) primers were applied for sequencing in addition to the primers used for primary PCR to assure that both nucleotide directions were covered [22]. The BioEdit (version 7.0.5.3) program was used for the alignment and manual editing of acquired sequences. The cap contig function of the BioEdit sequence editor (version 7.0.5.3) was used to build the consensus sequences, which were compared with those in the EzTaxon database [35] using the FASTA search tool to calculate the pairwise 16S rRNA gene sequence. 

### 3.4. Bacterial Extract Preparation and Antimicrobial Assay 

Well-grown cultures of sponge-associated isolates on agar plates (containing 3.00 g of casitone, 1.36 g of CaCl_2_·2H_2_O, 1.00 g of yeast extract, 16.00 g of agar supplemented with 39 g of artificial sea salts, and 1000 mL of distilled water, with the pH adjusted to 7.2 before sterilization) were cut into small pieces (1 cm), and three pieces per flask were inoculated in 250 mL Erlenmeyer flasks containing 100 mL of marine broth medium at 30 °C with 160 rpm agitation for 4 days. After incubation, 25 mL of cell cultures were mixed with 25 mL of ethyl acetate in 50 mL falcon tubes. The tubes were mixed for 20 min in a rotary shaker and then centrifuged at 9000 rpm for 10 min; then, the organic phase was separated. The biomass was extracted with ethyl acetate three times, and the organic phases were combined in a 50 mL round-bottom flask. At 40 °C, the ethyl acetate was evaporated in a rotary evaporator to get a solid residue. Finally, the extract was dissolved in methanol to adjust to the concentration of 1 mg/mL and stored at −20 °C.

The marine-sponge-associated isolates were screened for antimicrobial activity using a minimum inhibitory concentration assay. The indicator strains used in the assay were *Escherichia coli* acrB JW25113, *Staphylococcus aureus* (Newman), *Citrobacter freundii* DSM 30039, *Bacillus subtilis* DSM 10, *Mycobacterium smegmatis* ATCC 700084, *Pseudomonas aeruginosa* PA14, *Mucor hiemalis* DSM 2656, *Pichia anomala* DSM 6766, and *Candida albicans* DSM 1665. The strains were added to 20 mL of their respective growth media (MYC medium containing 1.0% phytone peptones, 1.0% glucose, and 50 mM Hepes at 11.9 g/L, with a pH of 7.0 for fungi and yeasts, and Mueller–Hinton broth medium containing 0.5% casein peptone, 0.1% meat extract, 0.5 % protease peptone, 0.1% yeast extract, with a pH of 7.0 for bacteria) [36]) and mixed well. Diluted cultures in a volume of 150 μL were transferred into each well of a 96-well plate (initial OD600 for the bacteria was 0.01, and for fungi and yeasts, it was 0.05). The first row was augmented by an additional 130 μL of indicator culture. Crude extracts (20 μL) of the isolated strains were added to the first row. The extract was serially diluted (1:1) by transferring 150 μL from one well to the next in a 96-well plate. Antibiotics and methanol were used as positive and negative controls, respectively. Thereafter, the microtiter plates were incubated on a microplate shaker incubator at 160 rpm at 30 or 37 °C for 24 h. Visual inspection of the indicator strain was performed after incubation, and the MIC values were recorded as the lowest concentrations of the substance that had no visible bacterial turbidity. All tests were performed in triplicate.

### 3.5. Bioassay-Guided Fractionation and Mass Spectrometric Analysis

The obtained crude extract was fractionated using reverse-phase HPLC (RP-HPLC) on an Agilent 1260 HPLC system equipped with an XBridge column (Agilent Technologies, CA, USA; C-18 3.5 μm, 2.1 mm × 100 mm, Waters). The fractions were collected every 30 s by the HPLC column in the 96-well plates and, finally, subjected to heated nitrogen for drying for 40 to 50 min at 40 °C. Afterwards, each well was filled with 150 μL of the susceptible strain and incubated for 24–48 h to locate the active fractions. 

The crude extract was subsequently separated and analyzed by using reverse-phase high-performance liquid chromatography (Agilent 1260 series) with diode-array detection and mass spectrometry (RP-HPLC-DAD-MS). High-resolution electron spray ionization mass spectrometry (HR-ESI-MS) data were generated with a Maxis ESI-TOF-MS spectrometer (Bruker, Fremont, CA, USA). The RP-HPLC system used Acquity C18 column 2.1 × 50 mm and 1.7 μm with gradient elution, employing two mobile phases (solvent A: water + 0.1 formic acid; solvent B: acetonitrile +0.1 formic acid) with a flow rate of 0.6 mL/min. The gradient system was 5% B in the first 0.5 min, increasing gradually to 100% B in 19.5 min, and, finally, staying for 5 min at 100% B. The ESI source was operated in the positive mode (4.5 kV, capillary voltage; 200 °C, drying gas temperature). Data processing for the identification of bioactive masses was carried out using the data analysis software included in the Compass software from Bruker. The chromatographic peaks obtained were identified by comparing them with those in the Dictionary of Natural Products database (https://dnp.chemnetbase.com/, accessed on 25 September 2021) [37]. 

### 3.6. Genome Sequencing, Functional Annotation, and Mining of the Novel Sponge Isolate

Genomic DNA for whole-genome sequencing was isolated using the Nucleospin Microbial DNA kit (Macherey-Nagel, Düren, Germany). Sequencing libraries were constructed using the Nextera XT library protocol kit, and sequencing was carried out using the Illumina MiSeq NGS platform (300 bp, paired-end reads) at the sequencing facility within the Helmholtz Center for Infection Research, Braunschweig, Germany. The high-quality NGS reads generated were assembled by employing the de novo approach of Unicycler [38]. The detailed in-depth subsystem and functional-role-based genome annotation were performed by using the RAST (Rapid Annotation using Subsystem Technology) algorithm (https://rast.nmpdr.org/, accessed on 11 October 2021) [39]. Biosynthetic gene clusters and secondary metabolites were predicted by using the online anti-SMASH software (https://antismash.secondarymetabolites.org/, accessed on 12 October 2021) [40]. Curated secondary metabolite biosynthetic gene clusters were reconstructed by submitting 54 assembled scaffolds (N50 = 385416) of *Qipengyuania pacifica* NZ-96^T^ to the anti-SMASH server. The anti-SMASH algorithm was used to predict the core genomic architecture of secondary metabolites that encoded biosynthetic gene clusters, and the functional attributes of all predicted genes in a biosynthetic gene cluster were curated in detail using cluster BLAST. 

## 4. Conclusions

To our knowledge, this is the first report to describe the bacterial population associated with the sponge species described in this study. The isolation and taxonomic characterization of the culturable sponge-associated bacterial communities depicted diverse putatively new taxa of *Alpha* and *Gamma proteobacteria* that need to be further characterized at the genome level. A good number of sponge-associated isolates were shown to be active against *B. subtilis* DSM 10 and *S. aureus* Newman, while fewer were found to be active against *Mycobacterium smegmatis* ATCC 700,084 and *Mucor hiemalis* DSM 2656. Pentadecenoic acid, oleic acid, and palmitoleic acid were tentatively identified as the compounds responsible for bioactivities. The novel isolate *Qipengyuania pacifica* NZ-96^T^ can be distinguished from the closely related types of strains by several striking characteristics based on genome analysis. The identification of bacteriocin (lassopeptide) and terpene BCGs in the genome of the novel isolate highlights its ability to produce antimicrobial compounds. Our findings show the biotechnological potential of sponge-associated bacteria, thus directing further research in the search for novel bioactive compounds.

## Figures and Tables

**Table 1:** Taxonomic affiliations of sponge-associated bacterial isolates.

Isolate	Closest Strain	Accession No.	Similarity	Genera	Phylum/Class
NZ-01	*Psychrobacter celer* SW-238	NR043225	99.81%	*Psychrobacter*	*Gammaproteobacteria*
NZ-02	*Mesonia mobilis* KMM 6059	NR043758	99.17%	*Mesonia*	*Bacteroidetes*
NZ-03	*Micromonospora chokoriensis* 2-19(6)	NR041349	99.42%	*Micromonospora*	*Actinobacteria*
NZ-04	*Zunongwangia profunda* SM-A87	NR074656	99.64%	*Zunongwangia*	*Bacteroidetes*
NZ-05	*Micromonospora chokoriensis* 2-19(6)	NR041349	99.32%	*Micromonospora*	*Actinobacteria*
NZ-06	*Alteriqipengyuania lutimaris* S-5	NR134028	98.65%	*Alteriqipengyuania*	*Alphaproteobacteria*
NZ-07	*Alteriqipengyuania lutimaris* S-5	NR134028	99.04%	*Alteriqipengyuania*	*Alphaproteobacteria*
NZ-08	*Paenisporosarcina quisquiliarum* SK55	NR043720	99.61%	*Paenisporosarcina*	*Firmicutes*
NZ-09	*Staphylococcus edaphicus* CCN 8730	NR156818	99.62%	*Staphylococcus*	*Firmicutes*
NZ-10	*Alteriqipengyuania lutimaris* S-5	NR134028	98.88	*Alteriqipengyuania*	*Alphaproteobacteria*
NZ-11	*Alteriqipengyuania lutimaris* S-5	NR134028	99.11%	*Alteriqipengyuania*	*Alphaproteobacteria*
NZ-12	*Zunongwangia profunda* SM-A87	NR074656	99.52%	*Zunongwangia*	*Bacteroidetes*
NZ-13	*Qipengyuania pelagi* UST081027-248	NR117936	99.02%	*Qipengyuania*	*Alphaproteobacteria*
NZ-14	*Psychrobacter submarinus* KMM225	NR025457	99.29%	*Psychrobacter*	*Gammaproteobacteria*
NZ-15	*Staphylococcus saprophyticus* NBRC46	NR114090	99.71%	*Staphylococcus*	*Firmicutes*
NZ-16	*Staphylococcus saprophyticus* NBRC46	NR114090	99.62%	*Staphylococcus*	*Firmicutes*
NZ-17	*Alteriqipengyuania lutimaris* S-5	NR134028	98.98%	*Alteriqipengyuania*	*Alphaproteobacteria*
NZ-18	*Qipengyuania pelagi* UST081027-248	NR117936	99.21%	*Qipengyuania*	*Alphaproteobacteria*
NZ-19	*Psychrobacter celer* SW-238	NR043225	99.23%	*Psychrobacter*	*Gammaproteobacteria*
NZ-20	*Psychrobacter submarinus* KMM225	NR025457	99.13%	*Psychrobacter*	*Gammaproteobacteria*
NZ-21	*Mesonia mobilis* KMM 6059	NR043758	99.15%	*Mesonia*	*Bacteroidetes*
NZ-22	*Halomonas profundus* AT1214	NR114956	98.58%	*Halomonas*	*Gammaproteobacteria*
NZ-23	*Qipengyuania citrea* 35F/1	NR028741	99.09%	*Qipengyuania*	*Alphaproteobacteria*
NZ-24	*Halomonas profundus* AT1214	NR114956	98.71%	*Halomonas*	*Gammaproteobacteria*
NZ-25	*Qipengyuania citrea* 35F/1	NR028741	99.63%	*Qipengyuania*	*Alphaproteobacteria*
NZ-26	*Halmonastitanicae* BH1	NR117300	98.82%	*Halomonas*	*Gammaproteobacteria*
NZ-27	*Halomonas titanicae* BH1	NR117300	98.64%	*Halomonas*	*Gammaproteobacteria*
NZ-28	*Halomonas titanicae* BH1	NR117300	98.77%	*Halomonas*	*Gammaproteobacteria*
NZ-29	*Alteriqipengyuania lutimaris* S-5	NR134028	99.23%	*Alteriqipengyuania*	*Alphaproteobacteria*
NZ-30	*Qipengyuania citrea* 35F/1	NR028741	99.62%	*Qipengyuania*	*Alphaproteobacteria*
NZ-31	*Microbacterium marytypicum* DSM12512	NR114986	99.43%	*Microbacterium*	*Actinobacteria*
NZ-32	*Mesonia mobilis* KMM 6059	NR043758	99.17%	*Mesonia*	*Bacteroidetes*
NZ-33	*Halomonas titanicae* BH1	NR117300	98.81%	*Halomonas*	*Gammaproteobacteria*
NZ-34	*Qipengyuania citrea* 35F/1	NR028741	99.72%	*Qipengyuania*	*Alphaproteobacteria*
NZ-35	*Qipengyuania citrea* 35F/1	NR028741	99.63%	*Qipengyuania*	*Alphaproteobacteria*
NZ-36	*Roseovarius nubinhibens* ISM	NR028728	100%	*Roseovarius*	*Alphaproteobacteria*
NZ-37	*Qipengyuania citrea* 35F/1	NR028741	99.53%	*Qipengyuania*	*Alphaproteobacteria*
NZ-38	*Roseovarius nubinhibens* ISM	NR028728	99.87%	*Roseovarius*	*Alphaproteobacteria*
NZ-39	*Maribacter dokdonensis* DSW-8	NR043294	99.71%	*Maribacter*	*Bacteroidetes*
NZ-40	*Qipengyuania citrea* 35F/1	NR028741	99.45%	*Qipengyuania*	*Alphaproteobacteria*
NZ-41	*Halomonas profundus* AT1214	NR114956	98.72%	*Halomonas*	*Gammaproteobacteria*
NZ-42	*Joostella marina* En5	NR044346	99.90%	*Joostella*	*Bacteroidetes*
NZ-43	*Pseudoalteromonas spongiae* UST723	NR043172	99.25%	*Pseudoalteromonas*	*Gammaproteobacteria*
NZ-44	*Bacillus hwajnpoensis* SW-72	AF541966	99.91%	*Bacillus*	*Firmicutes*
NZ-45	*Salinicola salarius* M27	NR042490	99.35%	*Salinicola*	*Gammaproteobacteria*
NZ-46	*Bacillus hwajnpoensis* SW-72	AF541966	99.72%	*Bacillus*	*Firmicutes*
NZ-47	*Pseudomonas zhaodongensis* STS-21	NR134795	99.32%	*Pseudomonas*	*Gammaproteobacteria*
NZ-48	*Joostella marina* En5	NR044346	99.85%	*Joostella*	*Bacteroidetes*
NZ-49	*Pseudoalteromonas spongiae* UST723	NR043172	99.01%	*Pseudoalteromonas*	*Gammaproteobacteria*
NZ-50	*Pseudoalteromonas spongiae* UST723	NR043172	99.25%	*Pseudoalteromonas*	*Gammaproteobacteria*
NZ-51	*Salegentibacter mishustinae* NBR100592	NR113918	99.59%	*Salegentibacter*	*Bacteroidetes*
NZ-52	*Halomonas axialensis* Althf1	NR027219	99.90%	*Halomonas*	*Gammaproteobacteria*
NZ-53	*Sulfitobacter pontiacus* ChLG-10	NR026418	99.61%	*Sulfitobacter*	*Alphaproteobacteria*
NZ-54	*Qipengyuania citrea* 35F/1	NR028741	99.56%	*Qipengyuania*	*Alphaproteobacteria*
NZ-55	*Tritonibacter mobilis* NBRC101030	NR041454	99.90%	*Tritonibacter*	*Alphaproteobacteria*
NZ-56	*Qipengyuania pelagi* UST081027-248	NR117936	98.86%	*Qipengyuania*	*Alphaproteobacteria*
NZ-57	*Bacillus hwajnpoensis* SW-72	AF541966	99.63%	*Bacillus*	*Firmicutes*
NZ-58	*Erythrobacter aureus* strain YH-07	NR169452	99.05%	*Erythrobacter*	*Alphaproteobacteria*
NZ-59	*Mesonia mobilis* KMM 6059	NR043758	99.26%	*Mesonia*	*Bacteroidetes*
NZ-60	*Sulfitobacter pontiacus* ChLG-10	NR026418	99.72%	*Sulfitobacter*	*Alphaproteobacteria*
NZ-61	*Sulfitobacter pontiacus* ChLG-10	NR026418	99.70%	*Sulfitobacter*	*Alphaproteobacteria*
NZ-62	*Tritonibacter mobilis* NBRC101030	NR041454	99.56%	*Tritonibacter*	*Alphaproteobacteria*
NZ-63	*Pseudoalteromonas tetraodonis* 458	NR114547	100%	*Pseudoalteromonas*	*Gammaproteobacteria*
NZ-64	*Tritonibacter mobilis* NBRC101030	NR041454	99.81%	*Tritonibacter*	*Alphaproteobacteria*
NZ-65	*Bacillus hwajnpoensis* SW-72	AF541966	99.74%	*Bacillus*	*Firmicutes*
NZ-66	*Marinobacter hydrocarbonoclasticus* ^RAD^	MH725589	99.81%	*Marinobacter*	*Gammaproteobacteria*
NZ-67	*Pseudoalteromonas spiralis* Te-2-2	NR114801	99.81%	*Pseudoalteromonas*	*Gammaproteobacteria*
NZ-68	*Qipengyuania citrea* 35F/1	NR028741	99.63%	*Qipengyuania*	*Alphaproteobacteria*
NZ-69	*Pseudomonas zhaodongensis* STS21	NR134795	99.35%	*Pseudomonas*	*Gammaproteobacteria*
NZ-70	*Pseudoalteromonas spiralis* Te-2-2	NR114801	99.77%	*Pseudoalteromonas*	*Gammaproteobacteria*
NZ-71	*Mesonia mobilis* KMM 6059	NR043758	99.42%	*Mesonia*	*Bacteroidetes*
NZ-72	*Kangiella japonica* KMM 3899	NR112923	99.41%	*Kangiella*	*Gammaproteobacteria*
NZ-73	*Psychrobacter submarinus* KMM 225	NR025457	99.29%	*Psychrobacter*	*Gammaproteobacteria*
NZ-74	*Qipengyuania citrea* 35F/1	NR028741	99.58%	*Qipengyuania*	*Alphaproteobacteria*
NZ-75	*Maribacter dokdonensis* DSW-8	NR043294	99.29%	*Maribacter*	*Bacteroidetes*
NZ-76	*Qipengyuania pelagi* UST081027-248	NR117936	99.04%	*Qipengyuania*	*Alphaproteobacteria*
NZ-77	*Kangiella japonica* KMM 3899	NR112923	99.63%	*Kangiella*	*Gammaproteobacteria*
NZ-78	*Joostella marina* En5	NR044346	99.91%	*Joostella*	*Bacteroidetes*
NZ-79	*Tritonibacter mobilis* NBRC101030	NR041454	99.92%	*Tritonibacter*	*Alphaproteobacteria*
NZ-80	*Halomonas axialensis* Althf1	NR027219	99.64%	*Halomonas*	*Gammaproteobacteria*
NZ-81	*Bacillus thuringiensis* IAM 12077	NR043403	99.71%	*Bacillus*	*Firmicutes*
NZ-82	*Psychrobacter celer* SW-238	NR043225	99.78%	*Psychrobacter*	*Gammaproteobacteria*
NZ-83	*Bacillus dakarensis* Marseille-P3515	NR147382	99.70%	*Bacillus*	*Firmicutes*
NZ-84	*Bacillus dakarensis* Marseille-P3515	NR147382	99.58%	*Bacillus*	*Firmicutes*
NZ-85	*Qipengyuania pelagi* UST081027-248	NR117936	99.12%	*Qipengyuania*	*Alphaproteobacteria*
NZ-86	*Dietzia kunjamensis* DSM 44907	NR116684	99.90%	*Dietzia*	*Actinobacteria*
NZ-87	*Dietzia kunjamensis* DSM 44907	NR116684	100%	*Dietzia*	*Actinobacteria*
NZ-88	*Zunongwangia profunda* SM-A87	NR074656	99.79%	*Zunongwangia*	*Bacteroidetes*
NZ-89	*Qipengyuania pelagi* UST081027-248	NR117936	99.16%	*Qipengyuania*	*Alphaproteobacteria*
NZ-90	*Psychrobacter celer* SW-238	NR043225	99.91%	*Psychrobacter*	*Gammaproteobacteria*
NZ-91	*Qipengyuania citrea* 35F/1	NR028741	99.01%	*Qipengyuania*	*Alphaproteobacteria*
NZ-92	*Zunongwangia profunda* SM-A87	NR074656	99.79%	*Zunongwangia*	*Bacteroidetes*
NZ-93	*Qipengyuania pelagi* UST081027-248	NR117936	99.22%	*Qipengyuania*	*Alphaproteobacteria*
NZ-94	*Halomonas titanicae* BH1	NR117300	98.52%	*Halomonas*	*Gammaproteobacteria*
NZ-95	*Maribacter sedimenticola* KMM 3903	NR025748	98.56%	*Maribacter*	*Bacteroidetes*
NZ-96	*Qipengyuania flava* SW-46	NR025245	98.30%	*Qipengyuania*	*Alphaproteobacteria*
NZ-97	*Bacillus dakarensis* Marseille-P3515	NR147382	99.23%	*Bacillus*	*Firmicutes*
NZ-98	*Tritonibacter mobilis* NBRC101030	NR041454	99.79%	*Tritonibacter*	*Alphaproteobacteria*
NZ-99	*Marinobacter hydrocarbonoclasticus* ^RAD^	MH725589	99.73%	*Marinobacter*	*Gammaproteobacteria*
NZ-100	*Halomonas axialensis* Althf1	NR027219	99.94%	*Halomonas*	*Gammaproteobacteria*
NZ-101	*Microbacterium marytypicum* 12512	NR114986	99.59%	*Microbacterium*	*Actinobacteria*
NZ-102	*Microbacterium marytypicum* 12512	NR114986	99.47%	*Microbacterium*	*Actinobacteria*
NZ-103	*Mesonia mobilis* KMM 6059	NR043758	99.25%	*Mesonia*	*Bacteroidetes*
NZ-104	*Psychrobacter celer* SW-238	NR043225	99.59%	*Psychrobacter*	*Gammaproteobacteria*
NZ-105	*Sulfitobacter pontiacus* ChLG-10	NR026418	99.67%	*Sulfitobacter*	*Alphaproteobacteria*

**Table 2:** Minimum inhibitory concentration values (µg/mL) with 1 mg/mL of crude extracts from representative bioactive genera. All compounds were dissolved in methanol (1 mg/mL, test volume: 20 μL). The amount of 20 μL of methanol showed no effect on the indicator organisms. Positive controls: Tetracycline, Nystatin, Gentamycin, and Kanamycin.

Test Organism	Minimum Inhibitory Concentration (MIC) (µg/mL)				
	*K. japonica*	*T. mobilis*	*P. zhaodongensis*	*Z. profunda*	Positive Control
*P. aeruginosa* PA14	>66.67	>66.67	>66.67	>66.67	0.52 ^3^
*B. subtilis* DSM 10	1.04	2.08	4.16	66.67	0.52 ^1^
*E. coli* DSM1116	>66.67	> 66.67	>66.67	>66.67	0.52 ^3^
*S. aureus* Newman	8.3	33.3	16.6	>66.67	0.52 ^3^
*M. smegmatis* ATCC700084	>66.67	>66.67	>66.67	66.67	0.52 ^4^
*C. freundii* DSM 30039	>66.67	>66.67	>66.67	>66.67	66.67
*C. albicans* DSM 1665	>66.67	>66.67	>66.67	>66.67	16.66 ^2^
*P. anomala* DSM 6766	>66.67	>66.67	>66.67	>66.67	8.33 ^2^
*M. hiemalis* DSM 2656	>66.67	>66.67	>66.67	66.67	16.6 ^2^

^1^ Tetracycline 10 mg/mL, ^2^ Nystatin 10 mg/mL, ^3^ Gentamycin 10 mg/mL, ^4^ Kanamycin 10 mg/mL.

**Table 3:** RAST-based curated subsystem and functional role counts of *Qipengyuania pacifica* NZ-96^T^ and closely related species.

Strains	Subsystem	Functional Roles
*Qipengyuania pelagi*	274	813
*Qipengyuania citreus*	272	803
*Qipengyuania pacifica* NZ-96^T^	282	877
Total	828	2493
Distinctive	308	973

**Table 4:** The putative BGCs predicted in *Qipengyuania pacifica* NZ-96^T^ using aniSMASH.

BGC	Position From-To	Size	Proposed Type (Known Product)
Cluster 1	298,580–326,894	28,315	Beta lactone
Cluster 2	112,027–136,358	24,332	Terpene (Zeaxanthin)
Cluster 3	35,697–73,244	37,548	Hserlactone, Lassopeptide

## Data Availability

Not applicable.

## Supplementary Materials

The following supporting information can be downloaded at: https://www.mdpi.com/article/10.3390/md20070463/s1, Figure S1: Chemical structures and features of pentadecenoic acid from *Kangiella japonica KMM 3899* (data from the Dictionary of Natural Product database). Figure S2: Chemical structures and features of 9-hexadecenoic acid and methyl ester from *Kangiella japonica KMM 3899* (data from the Dictionary of Natural Products database). Figure S3: Chemical structures and features of 9-octadecenoic acid from *Tritonibacter mobilis NBRC101030* (data from the Dictionary of Natural Products database). Figure S4: Chemical structures and features of methyl oleate from *Tritonibacter mobilis NBRC101030* (Data from the Dictionary of Natural Products database). Figure S5: Chemical structures and features of palmitoleic acid from *Pseudomonas zhaodongensis* NEAU-STS-21 (data from the Dictionary of Natural Products database). Table S1: Metabolism-related subsystem categories among *Q. pacifica* NZ-96^T^ and its closely related phylogenetic members. Table S2: Resistance to antibiotics and toxic compound subsystem categories among *Q. pacifica* NZ-96^T^ and its closely related phylogenetic members. Table S3: Basic local alignment search for NZ-96^T^ beta-lactone-like gene cluster (NZ-96^T^) displaying the highest sequence homology (~67.6%) with *Erythrobacter atlanticus* s21-N3. Table S4: Basic local alignment search for NZ-96^T^ terpene-like gene cluster showing the highest sequence similarity (~91.2%) with the *Erythrobacter flavus* strain KJ5. Table S5: Basic local alignment search for NZ-96^T^ Hserlactone|Lassopeptide-like gene cluster showing the highest sequence homology (~88% identity) with *Citromicrobium* sp. JL477.
