# Supplementary material for: Exploring the Antibiotic Production Potential of Heterotrophic Bacterial Communities Isolated from the Marine Sponges Crateromorpha meyeri, Pseudaxinella reticulata, Farrea similaris, and Caulophacus arcticus through Synergistic Metabolomic and Genomic Analyses
Source: Mar Drugs. 2022 Jul 20;20(7):463. doi: 10.3390/md20070463 (PMC9318849; doi:10.3390/md20070463)
Supplement: Supplementary file 1 [file marinedrugs-20-00463-s001.zip › marinedrugs-1809266-supplementary.pdf]

SUPPLEMENTARY INFORMATION

Exploring the Antibiotic Production Potential of Heterotrophic Bacterial Communities Isolated from the Marine Sponges *Crateromorpha meyeri, Pseudaxinella reticulata, Farrea similaris,* and *Caulophacus arcticus* through Synergistic Metabolomic and Genomic Analyses

Sanaullah Tareen [1,2], Peter J. Schupp [3], Naveed Iqbal [4] and Joachim Wink [1,*]

[1] Microbial Strain Collection (MISG), Helmholtz Centre for Infection Research (HZI), 38124 Braunschweig, Germany; sanaullah.tareen@helmholtz-hzi
[2] Department of Microbiology, Balochistan University of Information Technology Engineering
and Management Sciences, Quetta 87300, Pakistan
[3] Institute for Chemistry and Biology of the Marine Environment, Oldenburg University, 26129 Oldenburg, Germany; peter.schupp@uni-oldenburg.de
[4] Department of Biotechnology, Balochistan University of Information Technology Engineering
and Management Sciences, Quetta 87300, Pakistan; naveed.iqbal@buitms.edu.pk
* Correspondence: joachim.wink@helmholtz-hzi.de

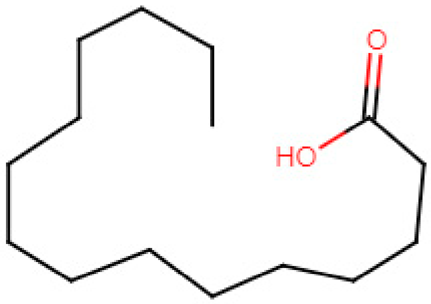

Entry Name: Pentadecanoic acid

Synonym(s): FEMA 4334.

Linear Diagram: $H_3C(CH_2)_{13}COOH$

CRC Number: BRH03-J

CAS Registry Number: 1002-84-2

Type of Compound Code(s): V.A.03000 W.A.20000 W.E.90000 W.I.50000 Z.Q.03200 Z.Q.26800 Z.Q.59000 Z.T.60000

Molecular Formula: $C_{15}H_{30}O_2$

Molecular Weight: 242.403

Accurate Mass: 242.22458

Percentage Composition: C 74.32%; H 12.48%; O 13.20%

Biological Source: Isol. from some plants, e.g. *Zanthoxylum carolinianum*, *Mallotus barbatus*, *Mangifera indica*. Occurs in lichens. Present in lipids of *Physalia physalis*. Minor component of mammalian milk

Use / Importance: Flavour and fragrance ingredient

Biological Use / Importance: Inhibitor of steroidal 5α-reductase

Physical Description: Cryst. (Me_2CO aq.) with waxy odour

Melting Point: Mp 53°

Figure S1. Chemical structures and features of Pentadecenoic acid from *Kangiella japonica KMM 3899* (Data from the dictionary of natural product database)

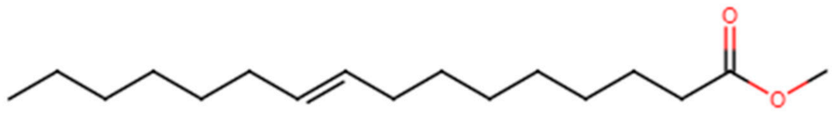

9-Hexadecenoic acid, methyl ester

Derivative: Me ester

CRC Number: HJM06-V

CAS Registry Number: 1120-25-8

Molecular Formula: $C_{17}H_{32}O_2$

Molecular Weight: 268.441

Accurate Mass: 268.24023

Percentage Composition: C 76.06%; H 12.02%; O 11.92%

Physical Description: Liq.

Boiling point: Bp_5 140- 141°

Aldrich: 36031-7

Fluka: 76176

Sigma: P6087

SMILES: CCCCCC\C=C\CCCCCCCC(=O)OC

InChi Key: InChIKey=IZFGRAGOVZCUFB-CMDGGOBGSA-N

InChi: InChI=1S/C17H32O2/c1-3-4-5-6-7-8-9-10-11-12-13-14-15-16-17(18)19-2/h8-9H,3-7,10-16H2,1-2H3/b9-8+

Figure S2. Chemical structures and features of 9-Hexadecenoic acid, methyl ester from *Kangiella japonica KMM 3899.* (Data from the dictionary of natural product database).

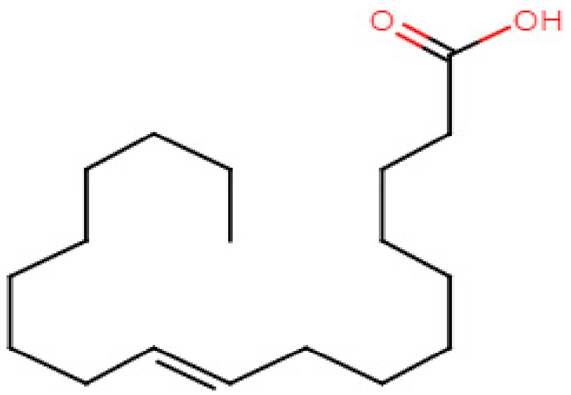

Entry Name: 9-Octadecenoic acid, CAS

Linear Diagram: $H_3C(CH_2)_7CH=CH(CH_2)_7$
COOH

CRC Number: BGT64-L

CAS Registry Number: 2027-47-6

Type of Compound Code(s): V.A.06000 Z.T.60000

Molecular Formula: $C_{18}H_{34}O_2$

Molecular Weight: 282.468

Accurate Mass: 282.25588

Percentage Composition: C 76.54%; H 12.13%; O 11.33%

Biological Source: Present in lipids of *Physalia physalis* (Portuguese-man-of-war)

SMILES: CCCCCCCC\C=C\CCCCCCCC(O)=O

InChi Key: InChIKey=ZQPPMHVWECSIRJ-MDZDMXLPSA-N

InChi: InChI=1S/C18H34O2/c1-2-3-4-5-6-7-8-9-10-11-12-13-14-15-16-17-18(19)20/h9-10H,2-8,11-17H2,1H3,(H,19,20)/b10-9+

Figure S3. Chemical structures and features of 9-octadecenoic acid from *Tritonibacter mobilis NBRC101030.* (Data from the dictionary of natural product database).

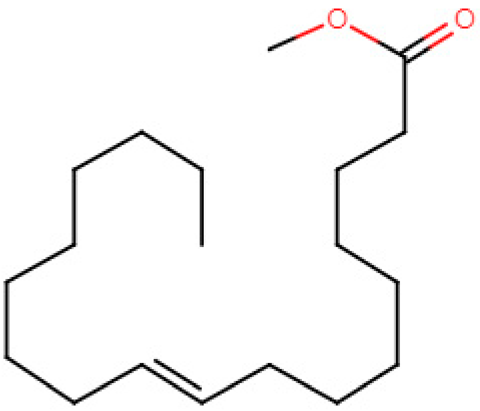

Methyl oleate

Derivative: Me ester

Synonym(s): Methyl oleate.

CRC Number: HMN61-I

CAS Registry Number: 112-62-9

Molecular Formula: $C_{19}H_{36}O_2$

Molecular Weight: 296.495

Accurate Mass: 296.27153

Percentage Composition: C 76.97%; H 12.24%; O 10.79%

Physical Description: Oil

Boiling point: Bp_{15} 212- 213°

Rare Chemicals Library: S42831-0

Hazard and Toxicity: An exp. carcinogen (v. large dose)

RTECS Accession Number: RK0895000

Aldrich: 26803-8

Fluka: 75162

Sigma: O1000

Supelco: R42-0555

SMILES: CCCCCCCC\C=C\CCCCCCCC(=O)OC

Figure S4. Chemical structures and features of Methyl oleate from *Tritonibacter mobilis NBRC101030.* (Data from the dictionary of natural product database).

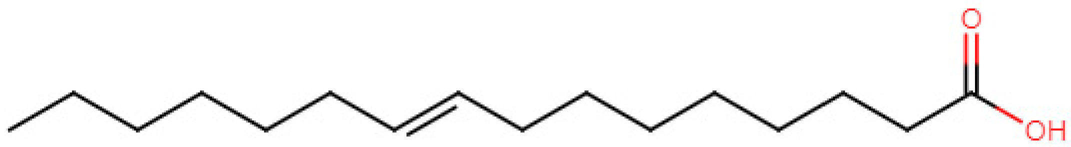

Palmitoleic acid

Entry Name: 9-Hexadecenoic acid, CAS

Synonym(s): Hypogaic acid.

Linear Diagram: $H_3C(CH_2)_5CH=CH(CH_2)_7$
COOH

CRC Number: CQD88-W

CAS Registry Number: 2091-29-4

Type of Compound Code(s): V.A.06000 Z.T.60000

Molecular Formula: $C_{16}H_{30}O_2$

Molecular Weight: 254.414

Accurate Mass: 254.22458

Percentage Composition: C 75.54%; H 11.89%; O 12.58%

Biological Source: Present in lipids of *Physalia physalis* (Portuguese-man-of-war)

SMILES: CCCCCC\C=C\CCCCCCCC(O)=O

InChi Key: InChIKey=SECPZKHBENQXJG-BQYQJAHWSA-N

InChi: InChI=1S/C16H30O2/c1-2-3-4-5-6-7-8-9-10-11-12-13-14-15-16(17)18/h7-8H,
2-6,9-15H2,1H3,(H,17,18)/b8-7+

Figure S5. Chemical structures and features of palmitoleic acid from *Pseudomonas zhaodongensis* NEAU-STS-21. (Data from the dictionary of natural product database).

Table S1: Metabolism-related subsystem categories among *Q. pacifica* NZ-96^T and its closely related phylogenetic members.

Metabolism related subsystem categories			
Protein Metabolism	190	177	180
DNA Metabolism	65	69	68
RNA Metabolism	30	28	28
Phosphorus Metabolism	24	21	23
Sulfur Metabolism	10	7	7
Nitrogen Metabolism	9	9	10
Potassium metabolism	7	9	8
Metabolism of Aromatic Compounds	6	16	7
Secondary Metabolism	4	4	4
Iron acquisition and metabolism	1	0	1
	346	340	336

Table S2: Resistance to antibiotics and toxic compounds subsystem categories among *Q. pacifica* NZ-96^T and its closely related phylogenetic members.

Subsystems for "Resistance to antibiotics and toxic compounds"			
	NZ-96^T	Qipengyuania pelagi JCM 17468	Qipengyuania citrea CGMCC 1.8703
Copper homeostasis	15	13	24
Cobalt-zinc-cadmium resistance	7	7	10
Resistance to fluoroquinolones	2	2	2
Copper homeostasis: copper tolerance	3	0	0
Beta-lactamase	1	1	1
Resistance to chromium compounds	1	2	0
Multidrug Resistance Efflux Pumps	3	3	1
Mercury resistance operon	0	2	1
Mercuric reductase	0	1	1
Invasion and intracellular resistance	12	12	12
Total	44	43	52

Table S3: Basic local alignment search for NZ-96^{T}-betalactone-like gene cluster (NZ-96T) displayed highest sequence homology (~67.6%) with *Erythrobacter atlanticus* s21-N3.

Query gene	*Subject gene*	*Identity (%)*	*Blast score*	*Coverage (%)*	*e-value*
qpaBTL-A	CP97_RS03070	82.4	238	98.62	7.3E-78
qpaBTL-B	CP97_RS03095	78	860	98.90	4.84e-313
qpaBTL-C	CP97_RS03100	81	746	99.31	1.3E-271
qpaBTL-D	CP97_RS03105	77	256	100.00	4.5E-87
qpaBTL-E	CP97_RS03110	74	589	99.24	5.1E-211
qpaBTL-F	CP97_RS16270	47	79	52.99	3.4E-18
qpaBTL-G	CP97_RS03120	73	833	96.76	3.5E-302
qpaBTL-H	CP97_RS03150	52	296	93.99	6.4E-99
qpaBTL-L	CP97_RS03130	61	303	94.07	2.3E-102
qpaBTL-L	CP97_RS03145	33	111	75.56	1.1E-27
qpaBTL-M	CP97_RS03135	65	371	97.12	1.9E-127
qpaBTL-N	CP97_RS03140	76	531	99.42	7.5E-190
qpaBTL-O	CP97_RS03145	63	256	84.38	1.4E-84
qpaBTL-X	CP97_RS03075	80.2	1291	98.49	0.0E+00
qpaBTL-Y	CP97_RS03085	71	161	83.19	3.3E-51
Average		**67.57**	**461.4**	**91.47**	**2.6E-19**

Table S4: Basic local alignment search for NZ-96T-terpene-like gene cluster showed highest sequence similarity (~91.2%) with the *Erythrobacter flavus* strain KJ5.

Query gene	***Subject gene***	***Identity (%)***	***Blast score***	***Coverage (%)***	***e-value***
QpaTrp-A	EKJ_RS04395	90	478	100.00	1.66E-171
QpaTrp-B	EKJ_RS04400	75	238	99.47	8.02E-79
QpaTrp-C	EKJ_RS04405	84	267	100.00	1.24E-91
QpaTrp-D	EKJ_RS04410	80	341	100.00	5.10E-119
QpaTrp-E	EKJ_RS04415	92	199	97.44	1.17E-64
QpaTrp-F	EKJ_RS04420	90	662	97.32	2.23E-240
QpaTrp-G	EKJ_RS04425	96	676	100.00	3.03E-244
QpaTrp-H	EKJ_RS04430	90	924	99.81	0.00E+00
QpaTrp-I	EKJ_RS04435	92	1108	100.00	0.00E+00
QpaTrp-J	EKJ_RS04440	96	602	98.50	2.37E-218
QpaTrp-K	EKJ_RS04445	95	376	97.97	3.02E-133
QpaTrp-L	EKJ_RS04450	90	940	96.68	0.00E+00
QpaTrp-M	EKJ_RS04455	99	636	99.49	1.79E-229
QpaTrp-N	EKJ_RS04460	95	374	100.35	1.68E-129
QpaTrp-O	EKJ_RS04465	84	704	100.00	1.64E-255
QpaTrp-P	EKJ_RS04470	76	926	99.84	0.00E+00
QpaTrp-Q	EKJ_RS04475	69	496	99.73	1.23E-174
QpaTrp-R	EKJ_RS13770	83	86	95.92	5.07E-23
QpaTrp-S	EKJ_RS04480	81	800	100.00	1.65E-290
QpaTrp-T	EKJ_RS04485	88	114	100.00	1.23E-33
QpaTrp-U	EKJ_RS04490	96	340	100.00	1.24E-119
Average		91.2%%	537	99.11%	2.41E-24

Table S5*:* Basic local alignment search for NZ-96^{T}-Hserlactone|Lassopeptide like gene cluster showed highest sequence homology (~88% identity) with *Citromicrobium* sp. JL477.

Query gene	***Subject gene***	***Identity (%)***	***Blast score***	***Coverage (%)***	***e-value***
QpaHsLas-A	WG74_RS01490	92	114	100.9	6E-31
QpaHsLas-B	WG74_RS01485	79	472	99.8	2E-159
QpaHsLas-C	WG74_RS01480	56	789	98.0	6E-280
QpaHsLas-D	WG74_RS01475	64	313	100.0	2E-100
QpaHsLas-E	WG74_RS01475	61	600	88.1	1E-206
QpaHsLas-F	WG74_RS01470	94	351	93.7	3E-123
QpaHsLas-G	WG74_RS16120	79	386	88.3	1E-134
QpaHsLas-H	WG74_RS01460	100	514	100.0	3E-186
QpaHsLas-I	WG74_RS01455	96	208	100.0	2E-69
QpaHsLas-J	WG74_RS01450	90	283	100.0	6E-98
QpaHsLas-K	WG74_RS01445	99	335	100.0	7E-118
QpaHsLas-L	WG74_RS01440	100	652	100.0	4E-237
QpaHsLas-M	WG74_RS01435	100	221	100.0	1E-74
QpaHsLas-N	WG74_RS01430	100	894	100.0	0E+00
QpaHsLas-O	WG74_RS15850	100	179	100.0	4E-59
QpaHsLas-P	WG74_RS01425	100	213	100.0	3E-71
QpaHsLas-Q	WG74_RS01420	100	240	100.0	6E-82
QpaHsLas-R	WG74_RS15845	99	282	100.0	8E-98
QpaHsLas-S	WG74_RS01410	100	253	100.0	1E-86
QpaHsLas-T	WG74_RS01405	100	726	100.0	2E-266
QpaHsLas-U	WG74_RS01400	100	427	100.0	4E-153
QpaHsLas-V	WG74_RS01395	100	599	100.0	1E-218
QpaHsLas-W	WG74_RS01390	99	394	100.0	3E-140
QpaHsLas-X	WG74_RS01385	100	830	100.0	9E-306
QpaHsLas-Y	WG74_RS01380	79	392	101.7	2E-138
QpaHsLas-Z	WG74_RS01370	63	491	99.0	1E-171
QpaHsLas-a	WG74_RS01365	98	451	100.0	1E-161
	Average	**88.0**	**430.0**	**98.9**	**2.21E-32**
